# Building-up a Smile in a 5-Year-Old Child: A Case Report

**DOI:** 10.5005/jp-journals-10005-1156

**Published:** 2012-08-08

**Authors:** Mohita Marwaha, Manohar Bhat, Kanwar Deep Singh Nanda

**Affiliations:** Senior Lecturer, Department of Pedodontics and Preventive Dentistry SGT Dental College, Hospital and Research Institute, Gurgaon Haryana, India, e-mail: dr.mohitamalhotra@gmail.com; Professor and Head, Department of Pedodontics, Jaipur Dental College, Jaipur, Rajasthan, India; Senior Lecturer, Department of Oral Pathology, SGT Dental College Hospital and Research Institute, Gurgaon, Haryana, India

**Keywords:** Fiber-splint, Replacement, Space maintainer

## Abstract

A variety of therapeutic modalities, from removable partial dentures to conventional fixed space maintainer can be used for the replacement of traumatically missing or carious lost primary anterior teeth. Dentistry has advanced to a point where it is undesirable for children to be partially edentulous or to have unattractive anterior teeth. The introduction of new materials and adhesive systems in dentistry, offers a new reconstructive alternative for severely destroyed or lost primary anterior teeth. The purpose of this article was to present a clinical case of four primary anterior teeth replacement by means of fiber-reinforced composite bridge. This technique offers a conservative, esthetic and noninvasive treatment. It can be considered, as a long- lasting reversible provisional treatment.

**How to cite this article:** Marwaha M, Bhat M, Nanda KDS. Building-up a Smile in a 5-Year-Old Child: A Case Report. Int J Clin Pediatr Dent 2012;5(2):151-154.

## INTRODUCTION

The restoration of primary maxillary incisors severely destroyed by trauma or caries, is a challenge for pediatric dentist. Esthetic restoration of primary anterior teeth can be especially challenging due to the small size of teeth, close proximity of pulp to tooth surface, relatively thin enamel and surface area for bonding, issues related to child behavior and finally cost of treatment.^1^ For the reconstruction or replacement of these primary anterior teeth, it is important to choose a material that is inexpensive, can be placed in one visit, and has the longevity to remain in place until just prior to the eruption of succedaneous teeth without interfering with the normal eruption process. In the modern civilized cosmetically conscious world, well contoured and well aligned white teeth set the standard for beauty. Such teeth are not only considered attractive, but are also indicative of nutritional health, self-esteem, hygiene pride and economic status.^2^

Various esthetic options are available for restoring or replacing the primary incisors and it depends upon the clinician to make the best choice of selection for each individual situation. For replacement of primary anterior teeth, the partial removable dentures are often recommended for very young patients depending upon the patient's compliance. These dentures could be modified when necessary by adding or grinding the acrylic resin. The replacement of missing primary maxillary anterior teeth can be made via fiber-reinforced composite bridge – an alternative to removable partial denture.

## CASE REPORT

A 5-year-old boy, reported to the department of pedodontics, with a chief complaint of pain in the lower right posterior tooth and the parents were also concerned about the esthetics too, and asked for the replacement of upper primary anterior teeth.

**Fig. 1:**
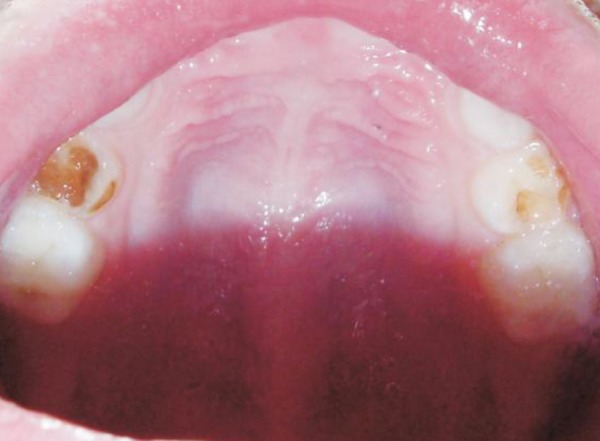
Preoperative maxillary arch

**Fig. 2:**
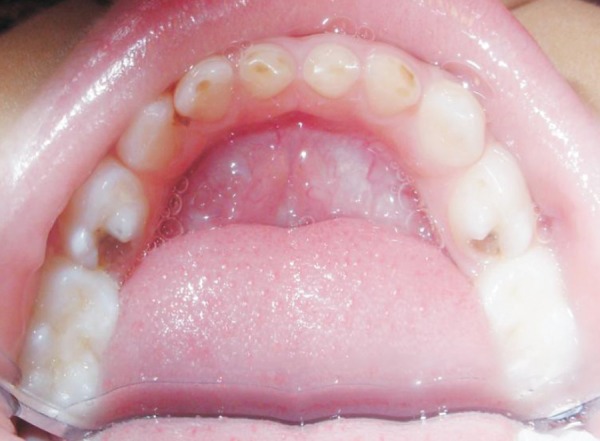
Preoperative mandibular arch

**Fig. 3:**
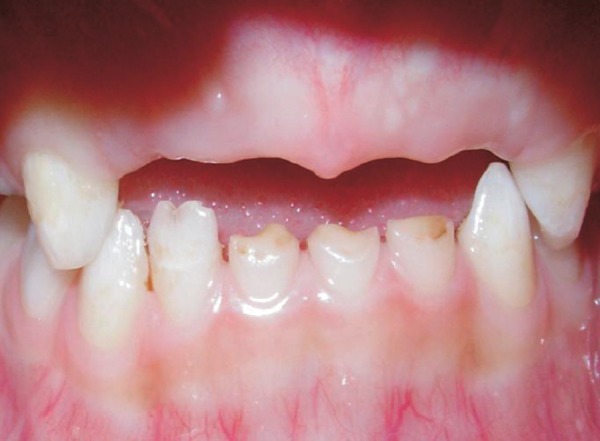
Preoperative: Teeth in occlusion

The boy's medical history revealed no specific problem. His past dental history indicated extraction of maxillary deciduous centrals and lateral incisors (51, 52, 61 and 62) due to caries. The clinical examination revealed carious maxillary right and left first deciduous molars (54 and 64), mandibular deciduous left canine (73), mandibular deciduous left and right first molars (74 and 84) and second molars (75 and 85) and attrited mandibular deciduous incisors (71, 72, 81 and 82) as shown in Figures 1 to 3.

### Radiographic Evaluation

Intraoral periapical radiograph were taken and revealed deep caries in relation to 54, 64, 74 and 84 pulp therapy was planned for the same followed by stainless steel crowns.

### Treatment Procedure

Intentional pulp therapy was done for attrited 71, 72, 81 and 82 followed by composite build-ups. Caries excavation was done in 73, 75 and 85 restored with glass ionomer cement. An impression was made with alginate for fiber-reinforced composite bridge formation to replace the missing 51, 52, 61 and 62. In this case, it was necessary to take a full-arch impression because there was a need to have the opposite model and proceed with articulating technique. The impression was poured with die material and the casts were articulated.

All the four teeth to be replaced were made in composite and to obtain good natural esthetics, a composite restorative system containing different enamel and dentin shades (Synergy D6 composite, Coltene Whaledent, Switzerland) was used.

After checking the occlusion with the anterior teeth in place, they were attached to each-other with the help of thin stainless steel wire (23 gauge) by creating a lingual groove on all four teeth and filling it with composite. These composite teeth together was to serve as a pontic.

The length measurement of fiber splint (Polydentia SA, Switzerland) was constructed with a thin wire. Wire was closely adapted to the working cast and was extended to the middle third of each abutment and crossed the pontic area directly under incisal edge. The wire was flattened and was used as a pattern, against which the exact length of fiber needed was measured.

One should avoid touching the fiber splint until after it is wetted with bonding resin (3M ESPE, Adper ^TM^ Single Bond 2, USA) via the fingers, because any contact can contaminate its reactive surface layer.

After recording the exact length, proper isolation of teeth was done with cotton rolls and enamel surface was etched with etchant (Totaletch, Ivoclar Vivadent, Liechtenstein) on the lingual surface. After rinsing, the surface was air- dried, visually inspected for proper acid etching and covered by a layer of bonding agent and light-cured. The reinforcement material, on the sides to be adapted on abutment teeth was wetted with bonding agent and a thin layer of microhybrid restorative material was placed on the lingual side of abutment teeth in the patient's mouth. This composite acted as glue and held the fiber during its adaptation. Using instruments, the fiber was properly adapted and excess composite was removed before light curing. The second piece of fiber splint was used in pontic region at this stage and it was impregnated with bonding agent, then carried into patient's mouth. A small amount of composite was applied to the fiber, over it the pontic were placed in proper position and the fiber is pushed through the uncured composite layer from the lingual side in the interproximal area between composite teeth and then light-cured.

**Fig. 4:**
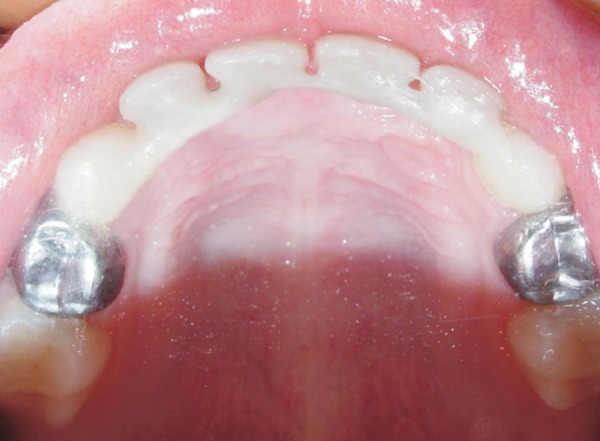
Postoperative: Fixed esthetic space maintainer replacing 51, 52, 61 and 62 stainless steel crowns cemented on 54 and 64

**Fig. 5:**
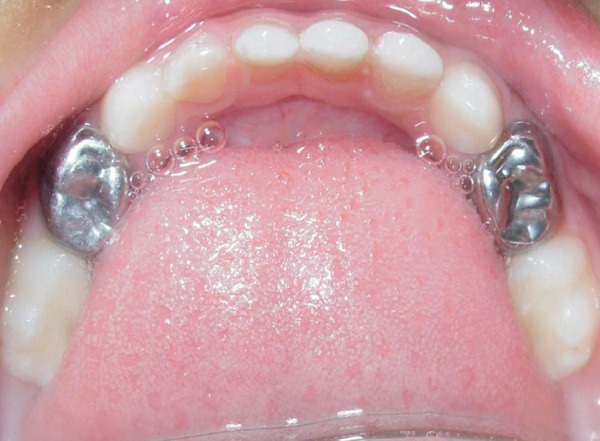
Postoperative: Stainless steel crowns cemented on 74 and 84 GIC restorations done in 75 and 85

**Fig. 6:**
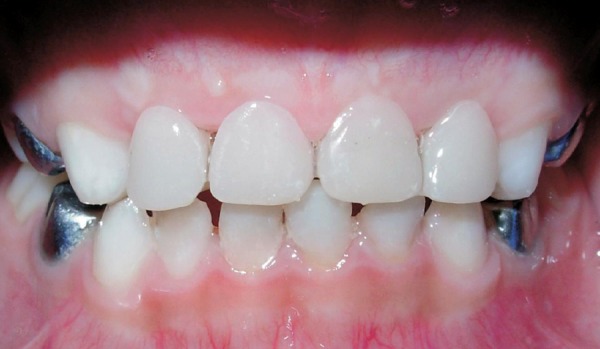
Postoperative: Teeth in occlusion

Once the bridge was placed, it was held firmly in position. Occlusion was checked at this before intraoral finishing and polishing. The final result was a well-adapted bridge with a natural esthetic result (Figs 4 to 6).

After 6 months, no problems appeared. The bonded space maintainer had immediately restored the esthetic functions and was well-accepted by the child.

## DISCUSSION

To avoid any malocclusion due to premature loss of primary teeth, clinician may advise various types of maintainers (removable or fixed appliances), depending upon the child's stage of dental development, the arch involved and the location of missing primary teeth.

Removable space maintainers have certain advantages, such as being easier to clean and allowing better maintenance of oral hygiene, they may be worn at whim of the patient and may be broken or lost easily and, if they are not used properly not be effective.^3^ In contrast, fixed appliances, if properly designed, are less damaging to the oral tissues and are worn continuously for a longer period.

The replacement of missing primary anterior teeth could be performed via different therapeutic options. Fiber- reinforced composite bridge, represent one of these options, with many advantages including bondability, ease of fabrication, reparability and relative longevity. This technique is considered as a noninvasive procedure and is easy to perform in pediatric patients.

The use of composites to build primary teeth provides a vital final aspect, with natural opalescence, translucency and opacity.

Most of the appliances used today maintain the arch length, but often disregard the functional aspect of the primary tooth.

Replacing the primary incisors with a bridge is a good option,^4^ but this solution is not realistic because the cost is too high, the teeth must be reduced, the adjacent tooth can be a permanent one and the chair time is too high for children. Fiber-reinforced composite bridge can be used as an alternative as it is less costly and labor intensive.^5^

A good space maintainer should present many qualities namely:^6^ Maintain space, prevent overeruption of antagonist, restore physiological mastication, allow for physiological maxillary growth, should be hygienic, have a good durability, have a low cost.

We presented a space maintainer showing most of these qualities. However, there is a disadvantage of this bonded bridge: If one of the support teeth is resorbed or exfoliated, the maintainer is lost. A clinical examination has to be made, to check the eruption of permanent teeth.

Fiber-reinforced composite's potential as a space maintainer in the primary or mixed dentition has gained popularity in the past years.^7^ These space maintainers, however, are often rigidly bonded to teeth, which may adversely influence the growth and development, exfoliation of primary teeth that the maintainers are attached to, and the eruption of succedaneous permanent teeth.^8^

These bridges represent an interesting alternative to conventional metal bridges.^9^ They could be directly or indirectly using an artificial plastic tooth or the avulsed tooth^5,10^ or by a direct build-up composite resin tooth with^5^ or without^11^ porcelain veneering.

The use of unreinforced composite resins as the structural material for bridges often results in fracture. Composite are brittle materials and contains bubbles, cracks, and other defects causing or facilitating fissure propagation and fracture.^12^ It has been demonstrated that the reinforcement of a composite resin by fibers increases the fracture toughness and resistance.^13^ The combination of an esthetic, wear- resistant composite resin, and tough fiber material gives a new option for short-span composite bridge fabrication.^5^

Whenever possible, a fiber-reinforced composite bridge should be fabricated extraorally to achieve better polish, polymerization conversion rate and adaptation.^5^

## CONCLUSION

1. Fiber-reinforced composite bridge fabrication technique presented in this article suggests a new treatment option for replacement of missing primary teeth.
2. This technique is more esthetic, function and comfortable than removable appliance.
3. It is easier to bond, more esthetically pleasing with no metal shadow.
4. It can be considered as a long-lasting provisional treatment.

## References

[1] Shah PV, Lee JY, Wright JT, (University of North Carolina School of Dentistry, The University of North Carolina at Chapel Hill, NC, USA) (2004). Clinical success and parental satisfaction with anterior preveneered primary stainless steel crowns.. Pediatr Dent.

[2] Arens D, (Indiana University). (1989). The role of bleaching in esthetics.. Dent Clin North Am.

[3] Subramaniam P, Babu G, Sunny R, (Department of Pedodontics and Preventive Dentistry, The Oxford Dental College, Hospital and Research Centre, Bommanahalli, Hosur Road, Bangalore-560 068, Karnataka, India. drpriyapedo@yahoo.com) (2008). Glass fiber-reinforced composite resin as a space maintainer: A clinical study.. J Indian Soc Pedod Prev Dent.

[4] Fortier JP, Ribes D (1981). Bridges anterieurs sur dents temporaries.. Act Odonto Stom.

[5] Chafaie A, Portier R, (Department of Paediatric Dentistry, Marseille, France. amir.chafaie@wanadoo.fr) (2004). Anterior fiber- reinforced composite resin bridge: A case report.. Pediatr Dent.

[6] Liegeois F, Limme M, (Department of Orthodontics and Pediatric Dentistry, Institut de Dentisterie, Liege, Belgium) (1999). Modified bonded bridge space maintainer.. J Clin Pediatr Dent.

[7] Kirzioglu Z, Eurturk MS, (Department of Pedodontics, Süleyman Demirel University, Isparta, Turkey. zuhal@med.sdu.edu.tr) (2004). Success of reinforced fiber material space maintainers.. J Dent Child (Chic).

[8] Kulkarni G, Lau D, Hafezi S, (Pediatric and Preventive Dentistry, Faculty of Dentistry, University of Toronto, Toronto, Ontario, Canada. g.kulkarni@utoronto.ca) (2009). Development and testing of Fiber-reinforced composite space maintainers.. J Dent Child (Chic).

[9] Vallittu PK, (Department of Prosthetic Dentistry and Biomaterials Research, Institute of Dentistry, University of Turku, Finland. pekka.vallittu@utu.fi) (2004). Survival rates of resin-bonded, glass-fiber-reinforced composite fixed partial dentures with a mean follow-up of 42 months: A pilot study.. J Prosthet Dent.

[10] Belli S, Ozer F, (Department of Operative Dentistry, Selçuk University, School of Dentistry, Campus, Konya, Turkey. b.sema@excite.com) (2000). A Simple method for single anterior tooth replacement.. J Adhes Dent.

[11] Van Wijlen P (2000). A modified technique for direct, fiber-reinforced, resin-bonded bridges: Clinical case reports.. J Can Dent Assoc.

[12] Rudo DN, Karbhari VM, (Department of Applied Mechanics and Engineering Sciences, University of California, San Diego, USA) (1999). Physical behaviors of fiber reinforcement as applied to tooth stabilization.. Dent Clin North Am.

[13] Pfeiffer P, Grube L, (Department of Prosthetic Dentistry, School of Oral Medicine, University of Cologne, Cologne, Germany. peter.pfeiffer@uni-koeln.de) (2003). In vitro resistance of reinforced interim fixed partial dentures.. J Prosthet Dent.

